# Concerns Regarding the Use of Dabigatran for Stroke Prevention in Atrial Fibrillation

**DOI:** 10.3390/ph5020155

**Published:** 2012-02-03

**Authors:** Claudia Stöllberger, Josef Finsterer

**Affiliations:** 1 Krankenanstalt Rudolfstiftung, Second Medical Department, Juchgasse 25, Wien A-1030, Austria; 2 Danube University Krems, Doktor-Karl-Dorrek-Straße 30, Krems A-3500, Austria; Email: josef.finsterer@wienkav.at

**Keywords:** atrial fibrillation, dabigatran, anticoagulation

## Abstract

Dabigatran is an oral thrombin inhibitor which has been approved in several countries as an alternative to vitamin-K-antagonists for the prevention of stroke or embolism in atrial fibrillation patients. Dabigatran is introduced into clinical practice, although many issues regarding this drug are still unclear, like laboratory monitoring, use in elderly patients, drug- and food-interactions and use in patients with renal insufficiency. Additionally, there is no antidote for dabigatran. Thus, aim of the present review is to give an overview of concerns and unresolved issues concerning dabigatran.

## 1. Introduction

In atrial fibrillation (AF), loss of mechanical atrial function and subsequent blood stasis predispose to thrombus formation and this increases the risk of stroke or peripheral embolism (S/E). In randomized clinical trials of AF-patients, vitamin-K-antagonists (VKA) decreased the risk of S/E compared with placebo by 64% [1]. Guidelines recommend VKA with an international normalised ratio (INR) range of 2.0–3.0 for AF-patients with an increased risk of S/E [2]. The risk of S/E is estimated by calculation of the CHADS_2_ or CHA_2_DS_2_VASc-scores (Table 1) [3,4].

**Table 1:** The CHADS_2_ and CHA_2_DS_2_VASc Scores [2].

Parameter		Score
**CHADS_2_**		
	Congestive heart failure	1
	Hypertension (blood pressure >140/90 mm Hg or treated hypertension on medication)	1
	Age ≥75 years	1
	Diabetes mellitus	1
	Stroke/transient ischemic attack/systemic embolism	2
	Maximum score	6
**CHA_2_DS_2_VASc**		
	Congestive heart failure	1
	Hypertension (blood pressure >140/90 mm Hg or treated hypertension on medication)	1
	Age ≥75 years	2
	Diabetes mellitus	1
	Stroke/transient ischemic attack/systemic embolism	2
	Vascular disease	1
	Age 65–74 years	1
	Sex category (female)	1
	Maximum score	9

Despite their efficacy, VKA are underused in AF-patients, especially in those who may benefit most, such as patients with advanced age or previous stroke [5,6]. If VKA are used, patients are within the therapeutic (INR) range only 50–60% of the time [7]. Reasons for the underuse of VKA include concerns about bleeding risk, frailty with a tendency to falls, interactions with other drugs and food, genetic polymorphisms affecting VKA metabolism, poor adherence to therapy and the need for frequent laboratory monitoring.

There is a general need for anticoagulant agents which overcome the obstacles of VKA by being effective, safe and convenient to use. Dabigatran is a new oral thrombin-inhibitor. The RE-LY trial, supported by Boehringer Ingelheim, was a noninferiority trial, in which fixed doses of dabigatran, 110 mg or 150 mg twice daily, were compared with adjusted-dose warfarin, a VKA, in 18,113 AF patients [8,9]. The duration of follow-up was 2.0 years. The primary outcome was stroke or systemic embolism. Yearly rates of S/E were 1.71% with warfarin, 1.54% with 110 mg dabigatran and 1.11% with 150 mg dabigatran. Yearly rates of major bleeding were 3.57% with warfarin, 2.87% with 110 mg dabigatran and 3.32% with 150 mg dabigatran. Yearly rates of hemorrhagic stroke were 0.38% with warfarin, 0.12% with 110 mg dabigatran and 0.10% with 150 mg dabigatran. Yearly mortality rates were 4.13% with warfarin, 3.75% with 110 mg dabigatran and 3.64% with 150 mg dabigatran. Dabigatran was thus suggested as a drug with a similar effect for stroke prevention as warfarin but with a lower complication rate [8].

Based on the results of the RE-LY-trial, dabigatran was approved in Canada and USA for stroke prevention in AF in November 2010. In April 2011 it was approved in this indication by the European Medicines Agency. The American Heart Association recommended dabigatran with a class 1, level of evidence B, as an alternative to warfarin for S/E-prevention in patients with paroxysmal or permanent AF who do not have severe renal failure (creatinine clearance <15 mL/min) or advanced liver disease [10]. Although not tested by randomized trials and not approved, dabigatran is already used in patients undergoing cardioversion as well as after AF-ablation [11,12].

Dabigatran is thus introduced into clinical practice, although many issues regarding this drug remain unclear, like laboratory monitoring, use in elderly patients, drug interactions and use in patients with renal insufficiency. Additionally, there is no antidote for dabigatran. Thus, aim of the present review is to give an overview of concerns and unresolved issues concerning dabigatran.

## 2. Methods

A literature search was carried out by systematically screening MEDLINE for publications with the key words “dabigatran” and “atrial fibrillation” from 2000 to 2011. Reference lists and older references generated from initial papers were also considered. Randomized clinical trials, longitudinal studies, case series, case reports and reports from regulatory agencies were also included.

## 3. Pharmacology of Dabigatran

Dabigatran etexilate is a prodrug that is given orally in a fixed dose and rapidly converted by cytochrome P 450-independent esterases to dabigatran, a potent reversible direct competitive inhibitor of thrombin with a rapid onset of action. The absolute bioavailability of dabigatran after oral application is 6.5% when the capsule is swallowed intact [13]. If the capsule shell is violated before ingestion, the oral bioavailability nearly doubles, thus the capsules must not be cut, chewed, or opened prior to ingestion. Dabigatran has a 35% plasma protein binding rate. Dabigatran is 85% excreted by the kidneys, and the plasma half-life is 12–17 h. It is assumed that the anticoagulant effect is predictable and consistent [14]. Pharmacokinetics and metabolism studies published so far come exclusively from laboratories of the manufacturer of the drug, and no independent studies, so far, have confirmed their findings [13,14].

## 4. Analysis of the Outcome Events in RE-LY

The outcome-events of the RE-LY trial were so far analyzed only according to the intention-to-treat-principle, an analysis which is based on the initial treatment intent but not on the treatment eventually administered [8]. Since the discontinuation rate was up to 21%, and higher in the group of patients treated with dabigatran than with warfarin, it is important to analyze the data also on a per-protocol-principle, an analysis in which only patients who actually complete the entire trial are counted towards the final results, and to assess if dabigatran was indeed associated with less embolic and bleeding events and a lower mortality compared with warfarin.

## 5. Dabigatran in Elderly Patients

Age-related differences in dabigatran exposure are largely related to renal function, although there is a small additional effect due to advancing age. Studies on the pharmacokinetics and pharmaco-dynamics of dabigatran in healthy elderly subjects indicate that, compared with young healthy subjects, the dabigatran bioavailability increases 1.7 to 2-fold in elderly subjects [15]. Since only mean values and standard deviations are reported, no information about individual measurement-variablities are available [15]. Data from the RE-LY trial confirm that dabigatran concentration is increased 1.3-fold in patients aged 65–75 years and 1.7-fold in those >75 years; these increases correspond to a decreased clearance of 0.66% for each year of age >68 years [8].

Among AF-patients >65 years, the most frequent reasons for not prescribing VKA are a history of bleeding (33%), falls (32%), refusal or nonadherence (14%), advanced illness (8%) and cognitive impairment (3%) [16]. We cannot find any data indicating that dabigatran might be the “drug of choice” for these patients. In contrast, the patients included in RE-LY were 71.5 years and thus younger than the majority of AF patients, only 32% had a CHADS_2_ score >2 and only 20% a creatinine clearance of <50 mL/min [8]. In RE-LY intracranial bleeding was less frequent with dabigatran at either dose than with warfarin at all ages. However, a subgroup analysis of the RE-LY trial showed that in patients ≥75 years, there was a trend that major bleeding was more frequent in patients under dabigatran than under warfarin [17]. In the meantime, several cases of elderly patients have been reported who, outside clinical trials, suffered from major bleeding events because of dabigatran accumulation, mainly attributable to renal dysfunction [18,19,20].

## 6. Dabigatran in Renal Insufficiency

Atrial fibrillation is frequently associated with chronic kidney disease, especially in elderly patients. In a Swedish registry for anticoagulation, 8% of AF-patients had an estimated glomerular filtration rate (GFR) <30 mL/min/1.73 m^2^ and 23% <45 mL/min/1.73 m^2^. GFR decreased with increasing age, and in patients ≥75 years, 11% had a GFR <30 mL/min/1.73 m^2^ [21]. After oral administration of a single dose of 150 mg dabigatran etexilate, its pharmacokinetic properties are affected by renal failure. Compared with the values in healthy subjects, the area under the curve (AUC) values for the plasma concentration-time were 1.5-, 3.2- and 6.3-fold higher in subjects with a creatinine clearance of 50–80 mL/min, 30–50 mL/min, and <30 mL/min respectively [22].

Patients with a creatinine clearance <30 mL/min were excluded from the RE-LY trial [8]. Thus, the recommendation of the American Heart Association to prescribe 75 mg dabigatran to patients with a creatinine clearance of 15–30 mL/min is surprising [10,23]. The evidence for this recommendation derives from pharmacokinetic modeling [24]. Dabigatran has not been tested in patients with severe renal failure, and neither the safety nor the efficacy of that dosage is known. 

## 7. Bleeding Complications

Since the rate of cerebral bleeding in the RE-LY trial was lower in dabigatran- than in warfarin-treated patients, it is recommended to treat patients with a history of intracranial bleeding with dabigatran rather than with VKA as a grade A recommendation [25]. However, cerebral bleeding was an exclusion criterion for the RE-LY trial, and thus, the RE-LY data cannot substantiate such a recommendation.

The rates of extra-cranial hemorrhage did not differ between dabigatran and warfarin-treated patients in the RE-LY trial. Yearly rates of extra-cranial bleeding were 2.84% with warfarin, 2.66% with 110 mg dabigatran and 3.02% with 150 mg dabigatran [8]. A subgroup analysis of the RE-LY trial showed that in patients ≥75 years, there was a trend that major bleeding was more frequent in dabigatran- than in warfarin-treated patients [17].

## 8. Dabigatran and Platelet Activation

In the PETRO-trial, which aimed to find a safe dose of dabigatran in patients with AF, urinary excretion of 11-dehydrothromboxane B2 (DTB2) was approximately 20% higher after 12 weeks of dabigatran treatment compared with warfarin in patients who did not receive acetylsalicylic acid [26]. This increase of thromboxane excretion suggests a platelet activating effect of dabigatran in the absence of concomitant acetylsalicylic acid. The authors of the trial conclude from this observation that “the significance of the increase of DTB2 concentrations in dabigatran-treated patients needs resolution”, which has, so far, not been accomplished. If the trend for a higher rate of myocardial infarction in dabigatran-treated (0.91 and 0.88%) *versus* 0.72% in warfarin-treated patients in the RE-LY trial may be related to the platelet-activating activity of dabigatran is unexplained [8]. A further explanation for the lower rate of myocardial infarction in VKA-treated patients might be that VKA are protective against myocardial infarction [27]. Concerns about cardiovascular side effects of dabigatran are further substantiated by a recently published meta-analysis comprising seven trials including 30,514 patients that reported acute coronary events as secondary outcomes. In that analysis dabigatran was associated with an increased risk of acute coronary events when tested against different controls [28]. The reason for this phenomenon is, so far, unknown.

## 9. Concerns Regarding Laboratory Monitoring

In certain clinical situations, such as emergency surgery, critical bleeding or when switching treatment from VKA to dabigatran or *vice versa*, clinicians need to determine the anticoagulant status of patients receiving dabigatran. Several coagulation tests can be used to assess the anticoagulant effect of dabigatran as listed in Table 2. The best tests to quantitatively evaluate the anticoagulant effects of dabigatran are the thrombin (TT) and ecarin clotting time, however the latter is not available in clinical practice [29]. The relationships between plasma concentrations of dabigatran and the TT, ecarin clotting time, and INR are linear. Dabigatran prolongs the TT, ecarin clotting time in a concentration-dependent manner for therapeutic concentrations, whereas the aPTT concentration-response curve is curvilinear and flattens at higher dabigatran concentrations (>200 ng/mL). The effect of dabigatran on INR is unpredictable. Thus, while measurement of aPTT may provide a qualitative indication of the anticoagulant activity, it is not suitable for the precise quantification of anticoagulant effect, especially at high plasma concentrations of dabigatran [30]. When using point-of-care INR devices, INR may be falsely elevated in dabigatran-treated patients [31]. A systematic, not manufacturer-related study in healthy patients on the effects of dabigatran on different coagulation assays found that different coagulation assays, display variable results at therapeutic concentrations of dabigatran [32]. Some of these assay variations are of clinical importance. Furthermore, the study showed that antithrombin assays based on thrombin inhibition but not Xa-inhibition give false high results, some assays for fibrinogen give correct results, other much too low results, and APC-resistance measurements are grossly affected [32]. Thus knowledge on effects of thrombin inhibitor effects is needed for a correct interpretation of results.

A dilute thrombin time assay (Hemoclot test, Hyphen Biomed, France) has been certified in several European countries for the determination of dabigatran plasma levels [29]. Unfortunately, no results about the validity of this test are published yet. Recently, the development of a liquid chromatography method, coupled with tandem mass spectrometry detection, has been reported for quantification of dabigatran in human plasma with [^13^C_6_]-dabigatran as internal standard [33]. It is uncertain, however, when this test will be available for clinical use. In conclusion, there are many unresolved issues regarding laboratory monitoring of the effects of dabigatran.

**Table 2:** Potentially useful test for the anticoagulant effect of dabigatran [27,55].

Characteristics	aPTT	PT	ECT	TT
Mechanism of markers	Intrinsic pathway	Extrinsic pathway	Activity of thrombin	Activity of thrombin
Response at recommended therapetic concentration of dabigatran	Linear	Less linear	Linear	Linear
Response at high concentration of dabigatran	Plateau effect	Less linear	Linear	Linear
Commercially available	Y	Y	N	Y

aPTT = activated partial thromboplastin time; PT = prothrombin time; ECT = Ecarin clotting time; TT = Thrombin time; Y = Yes; N = No.

Concerns about the lack of laboratory monitoring of emergency situations after trauma have been raised recently by American traumatologists [34].

## 10. Side Effects of Dabigatran

Rates of dyspepsia were 12% with dabigatran and 6% with warfarin and contributed to the high rate of 21% dropouts in the dabigatran-group in the RE-LY trial [8]. Drug-induced exanthema have been reported as further side effects of dabigatran [35,36,37]. Whether dabigatran will be accepted by patients in every-day life, and whether the compliance will be better with dabigatran than with warfarin is at present unknown and has to be investigated. Patients with low compliance seem no good candidates for dabigatran since it has to be taken twice daily whereas warfarin has to be taken only once daily.

## 11. Concerns about Patients’ Adherence

Although poor adherence was an exclusion criterion for the RE-LY trial, the discontinuation rate was high (21% for dabigatran, 17% for warfarin) during two years of follow-up [8]. Most of the discontinuations occurred because of dabigatran’s gastrointestinal side effects. It can be expected that outside clinical trials the discontinuation rate might be higher. Poor adherence in taking VKA can be easily detected by measuring the INR. Conversely, patients on dabigatran cannot be monitored by laboratory control easily, thus it is more difficult to evaluate patients’ adherence to dabigatran than to VKA. Special care has to be taken for the correct intake of the dabigatran capsules since they must not be chewed before ingestion, which is sometimes difficult to achieve in demented or non-adherent patients or patients with dental prostheses.

## 12. Safety and Efficacy in the Long Term (>2 Years Follow-Up)

Dabigatran has previously been used, and is approved for prophylaxis of venous thromboembolism, but the treatment duration was only up to four weeks. In AF, however, anticoagulation is necessary for many years. Thrombin is not only important for the coagulation system but plays also a role in infection, the immune response, angiogenesis, tumour growth and endothelial functions [37]. Whether the higher rate of myocardial infarction with dabigatran than with warfarin may be explained by long-term thrombin-inhibition has to be investigated. Furthermore, it would be interesting to compare the rate of cancer and infections among the 316 non-vascular deaths in the dabigatran-group with the 170 in the warfarin-group in the RE-LY trial [8].

## 13. No Antidote

No specific antidote is available to reverse the anticoagulant effects of dabigatran. However, dabigatran could be adsorbed by means of hemoperfusion over a charcoal filter. In case of major life-threatening bleeding, haemodialysis is another therapeutic option because of the relatively low (35%) plasma protein binding [20]. Additionally, application of recombinant activated factor VII (rFVIIa) reduced bleeding time and prolongation of aPTT associated with dabigatran in a rat tail model [29]. In a further animal experiment, prothrombin complex concentrate and, less consistently, fresh-frozen plasma prevented excess intracerebral hematoma expansion in dabigatran-treated mice with collagenase-induced intracerebral hemorrhages [38]. The clinical utility of these measures in humans taking dabigatran who are actively bleeding has not been established. In healthy humans, on the contrary, application of prothrombin complex concentrate had no influence on the coagulation parameters during treatment with dabigatran [39]. A further, so far unresolved issue is the decision about thrombolysis in a patient who develops an ischemic stroke under dabigatran. So far, only one case with atrial fibrillation and stroke under dabigatran with favourable outcome after thrombolysis has been reported [40].

## 14. Postoperative Use

In AF-patients, no data about postoperative use of dabigatran are available. Major surgery within the previous month was an exclusion criterion in the RE-LY trial [8]. Severe bleeding necessitating emergency dialysis was recently described in a patient in whom dabigatran was initiated on the 3rd postoperative day after coronary artery bypass graft surgery [20].

In a prospective study in 56 patients after hip arthroplasty, wound discharge after five days was significantly higher in patients taking dabigatran (32%) than in a historical group with dalteparin (10%), and the rate of delayed discharges due to wound discharge was 7% in the dalteparin group compared to 27% for dabigatran. Patients who received dabigatran were more than five times as likely to develop a wound complication compared with those who received dalteparin (7% dabigatran *vs.* 1% dalteparin) [41]. On the contrary, in the randomized RE-NOVATE II trial, sponsored by Boehringer-Ingelheim, in 2013 patients after hip arthroplaty no difference in postoperative insertion of drains or total wound drainage was observed between patients who received dabigatran *versus* enoxaparin [42].

## 15. Drug and Food Interactions via P-Glycoprotein-Affecting Drugs and Food Components

One of the advantages of dabigatran compared with VKA should be its lower rate of drug- and food interactions. However, dabigatran-absorption is dependent on the intestinal P-glycoprotein (P-gp)-system. P-gp is a product of the *MDR1* (multi drug resistance 1) gene and has considerable genetic heterogeneity [43]. P-gp activity is influenced by several drugs and food components (Table 3) [44,45].

Interactions between dabigatran and P-gp-affecting drugs have mainly been studied in Phase I trials in healthy volunteers. Verapamil and amiodarone elevated dabigatran concentrations by 50–60% and clarithromycin by 19% [46]. In a substudy of the RE-LY trial it was shown the influence of proton-pump inhibitors, amiodarone and verapamil on the bioavailabily of dabigatran was investigated and shown to be significant [14].

A survey among 100 hospitalized AF patients showed that 42% of hospitalized AF patients and 48% of VKA-receiving patients take P-gp-affecting drugs [47]. Although most of these drug-drug interactions should have only minor clinical consequences, more information about the relevance of drug- and food interactions is warranted before dabigatran is widely used for stroke prevention.

**Table 3:** Drugs, food components and herbs known to affect P-glycoprotein activity [44,45].

Drug	Drug
Amiodarone	Mefloquine
Amitriptyline	Mesylate
Amprenavir	Nelfinavir
Astemizole	Nicardipine
Bepredil	Nifedipine
Bromocriptine	Ofloxacin
Carvedilol	Perphenazine
Chlorpromazine	Probenecid
Clarithromycin	Progesterone
Clotrimazole	Propafenone
Colchicine	Propranolol
Cortisol	Quinidine
Cyclosporine	Reserpine
Desipramine	Rifampin
Dexamethasone	Ritonavir
Diethazine	Saquinavir
Diltiazem	Silymarin
Dipyridamol	Simvastatin
Disulfiram	Sirolimus
Doxepin	Tacrolimus
Dronedarone	Tamoxifen
Erythromycin	Terfenadine
Felodipine	Testosterone
Fluphenazine	Thiethylperazine
Haloperidol	Thioridazine
Imatinib	Trifluperazine
Imipramine	Troleandomycin
Indinavir	Valspodar
Itraconazole	Verapamil
Ketoconazole	Vinblastine
Levomepromazine	Vitamin E
Lovastatin	Yohimbine
**Food component, herb**	
Black pepper (*Piper nigrum*)	
Ginkgo (*Gingko biloba*)	
Ginseng (*Panax ginseng*)	
Grapefruit juice	
Licorice root	
Seville orange (*Citrus aurantium*)	
St. John’s Wort (*Hypericum perforatum*)	
Valerian (*Valeriana officinalis*)	

No studies so far have been reported about food interactions in dabigatran-treated patients. However, several food components are known to influence the P-pg system, thus it can be expected that food interactions will be discovered in the future [48].

## 16. Extraordinary Side Effects

Antifibrotic effects on lung fibroblasts is a further effect of dabigatran due to its thrombin-inhibiting properties [49,50]. Whether dabigatran will be established as therapeutic option in fibrotic diseases like scleroderma is uncertain. Furthermore, whether the antifibrotic effects of dabigatran may lead to side effects and complications during long-term therapy has not been investigated. An interesting observation has been reported from mice, infected with staphylococcus aureus, in whom dabigatran therapy prevented agglutination of the bacteria and lethal outcome of sepsis [51]. In a further animal experiment, dabigatran-induced inhibition of staphylothrombin reduced *S. aureus* virulence in *in vitro* and *in vivo* models [52].

## 17. Dabigatran as Alternative to VKA?

Patients who are already taking and tolerating VKA with good INR control may prefer to stay on VKA and not switch to dabigatran. Patients may be discouraged from a switch because dabigatran will need to be administered twice daily and has a greater risk of non-haemorrhagic side effects like dyspepsia which may increase the likelihood of drug discontinuation [53]. A net clinical benefit of the new oral anticoagulants dabigatran, rivaroxaban and apixaban compared with VKA was calculated in a modelling analysis, using data from the Danish National Patient registry and from clinical trials investigating the new anticoagulants [54]. The modelling analysis showed that when the risk of bleeding and stroke are both high, the new anticoagulants appear to have a greater net clinical benefit compared with VKA. The future will show if this beneficial effect, found in the model, will also occur in clinical practice. 

## 18. Conclusions

There is growing support for patience in transitioning our atrial fibrillation patients treated with VKA to a relative unknown dabigatran. All the concerns mentioned above need to be thoroughly discussed and solved before dabigatran can be recommended as a safe and reliable anticoagulant in AF-patients. The reputation not only of health authorities but also of the company producing the drug is at high risk before not all these open questions and concerns are solved. Studies carried out by company-independent institutions are warranted to ensure that design of studies and analysis and interpretation of data meet the general requirements to reliably demonstrate that the drug is in fact a progress compared to previous anticoagulant management. Those involved in these studies need to be unbiased and not affiliated to the producer or the authority of approval. Independent investigations may provide the chance to confirm that dabigatran is indeed superior to VKA. Similar issues may arise with other new anticoagulant drugs like the Xa-inhibitors rivaroxaban and apixaban. In view of the above mentioned extraordinary side effects, it cannot be excluded that dabigatran turns out to be a drug for indications other than anticoagulation.
